# Heimler Syndrome Caused by Novel *PEX6* Variants: Clinical and Genetic Characterization in a Saudi Cohort

**DOI:** 10.3390/genes17040360

**Published:** 2026-03-24

**Authors:** Basamat AlMoallem

**Affiliations:** 1 Department of Ophthalmology, College of Medicine, King Saud University, Riyadh 12372, Saudi Arabia; balmoallem@ksu.edu.sa; 2 Department of Ophthalmology, King Saud University Medical City (KSUMC), Riyadh 12372, Saudi Arabia

**Keywords:** Heimler syndrome, *PEX6*, mutation, retinal dystrophy, Usher syndrome, peroxisomal disorders

## Abstract

**Background:** Heimler syndrome (HS) is a rare autosomal recessive disorder representing the mildest end of the peroxisome biogenesis disorder spectrum. It is caused by hypomorphic mutations in peroxisomal assembly genes, most commonly *PEX1* and *PEX6*, and is characterized by sensorineural hearing loss, amelogenesis imperfecta, and retinal dystrophy. Due to phenotypic overlap with other inherited sensory disorders, particularly Usher syndrome, diagnosis of this condition is frequently delayed. **Methods:** We investigated two unrelated Saudi families presenting with congenital hearing loss and retinal dystrophy who were initially diagnosed with Usher syndrome. Detailed clinical evaluation, including comprehensive ophthalmologic and audiologic assessments, was performed. Whole-exome sequencing (WES) was conducted to identify the underlying genetic cause, followed by variant filtering and in silico pathogenicity prediction. **Results:** We identified a novel homozygous missense variant, p.Val97Gly (V97G), in the *PEX6* gene that co-segregated with the disease phenotype in both families. This variant was absent from major population databases, including dbSNP, the 1000 Genomes Project, ExAC, and gnomAD, and was predicted to be deleterious by multiple in silico prediction tools. Clinically, affected individuals presented with congenital sensorineural hearing loss, pigmentary retinal dystrophy with electrophysiological evidence of cone–rod dysfunction, enamel abnormalities consistent with amelogenesis imperfecta, and mild dysmorphic facial features, supporting a diagnosis within the Heimler syndrome spectrum. **Conclusions:** Our findings expand the mutational spectrum of *PEX6* and highlight Heimler syndrome as an important differential diagnosis in patients presenting with Usher-like phenotypes. To the best of our knowledge, this study represents the first report of the *PEX6* p.Val97Gly variant associated with Heimler syndrome in a Saudi population, underscoring the value of whole-exome sequencing for accurate diagnosis and genetic counseling in individuals with inherited sensory disorders.

## 1. Introduction

Heimler syndrome (HS; OMIM #234580, #616617) is a rare autosomal recessive disorder that represents the mildest phenotype within the spectrum of peroxisome biogenesis disorders (PBDs) [1]. PBDs result from defects in genes required for peroxisome assembly and function, leading to impaired peroxisomal metabolism and the disruption of multiple cellular pathways including lipid metabolism, reactive oxygen species detoxification, and the biosynthesis of essential metabolites [1]. At the severe end of the spectrum are Zellweger spectrum disorders (ZSDs), characterized by profound multisystem involvement including neurological impairment, hepatic dysfunction, craniofacial abnormalities, and early mortality due to near-complete loss of peroxisomal function [2]. In contrast, Heimler syndrome results from hypomorphic mutations that allow the partial preservation of peroxisomal activity, producing a considerably milder clinical phenotype and allowing survival into adulthood [3].

HS is most commonly associated with pathogenic variants in *PEX1* and *PEX6*, which encode members of the ATPase class of enzymes associated with diverse cellular activities (AAA-ATPase). These proteins play a critical role in recycling the peroxisomal import receptor *PEX5* during matrix protein import, a process essential for proper peroxisome biogenesis and maintenance [4]. The dysfunction of these proteins disrupts peroxisomal assembly and leads to impaired peroxisomal metabolic pathways. However, in HS, the mutations typically retain partial protein function, explaining the milder phenotype compared with classical ZSDs [3].

Clinically, Heimler syndrome is characterized by a distinctive triad of sensorineural hearing loss (SNHL), amelogenesis imperfecta, and retinal dystrophy, often accompanied by subtle ectodermal abnormalities such as nail dysplasia, leukonychia, or enamel hypoplasia [3]. Sensorineural hearing loss is typically a congenital or early-onset condition, and patients may require hearing aids or cochlear implantation. Dental abnormalities, particularly enamel hypoplasia or amelogenesis imperfecta, are frequently present but may be overlooked in early clinical assessments. Retinal involvement typically manifests as pigmentary retinopathy or cone–rod dystrophy, with progressive photoreceptor degeneration leading to night blindness and visual field constriction [5].

Because these features overlap with other inherited sensory disorders, particularly Usher syndrome, patients with HS are frequently misdiagnosed during early clinical evaluation. Both conditions share a combination of hearing impairment and retinal degeneration, which can complicate differential diagnosis in the absence of obvious dental or ectodermal findings [6]. Furthermore, biochemical markers of peroxisomal dysfunction including very long chain fatty acids (VLCFAs) may be normal or only mildly abnormal in individuals with HS, making clinical recognition particularly challenging [3]. As a result, molecular genetic testing has become essential for establishing a definitive diagnosis.

The advent of next-generation sequencing (NGS) technologies, particularly whole-exome sequencing (WES), has significantly improved the diagnostic yield for rare Mendelian disorders and has facilitated the identification of atypical or mild presentations of known genetic diseases [7]. Regarding inherited retinal disorders and syndromic hearing loss, WES enables the simultaneous interrogation of hundreds of candidate genes and has become an invaluable tool for resolving clinically ambiguous cases [8]. Moreover, genomic approaches have helped to clarify the phenotypic spectrum associated with *PEX1* and *PEX6* mutations, demonstrating that variants in these genes can produce a wide continuum of disease severity, ranging from classical Zellweger syndrome to mild phenotypes such as Heimler syndrome [3].

Although several cases of HS have been reported worldwide, the condition remains extremely rare, and its genetic and clinical spectrum continues to expand. Studies conducted in populations with high rates of consanguinity, such as those in the Middle East, have been particularly informative in identifying novel pathogenic variants and founder mutations in rare autosomal recessive disorders [9].

In this study, we aim to describe the clinical and genetic features in two unrelated patients from two unrelated Saudi families both descending from consanguineous background, who initially presented with congenital sensorineural hearing loss and retinal dystrophy suggestive of Usher syndrome.

## 2. Materials and Methods

### 2.1. Participants and Ethics

Two unrelated Saudi families were referred to the Ophthalmic Genetics Clinic at King Abdulaziz University Hospital, Riyadh, Saudi Arabia, for evaluation of congenital hearing loss and retinal dystrophy. Clinical and genetic investigations were conducted after obtaining written informed consent from all participating individuals and available family members. This study was conducted in accordance with the principles of the Declaration of Helsinki and was approved by the Institutional Review Board (IRB Project No. E-24-8700) of King Saud University.

### 2.2. Clinical Evaluation

Ophthalmologic Assessment

Two affected individuals were referred to the ophthalmology clinic at King Abdulaziz University Hospital, Riyadh, Saudi Arabia, with a history of congenital hearing loss and progressive night blindness, and they were initially suspected to have Usher syndrome. Both patients underwent a comprehensive ophthalmologic evaluation, including best-corrected visual acuity (BCVA) assessment, slit-lamp biomicroscopy, dilated fundus examination, and retinal imaging. Optical coherence tomography (OCT) was attempted; however, reliable scans could not be obtained because nystagmus resulted in poor fixation, limiting image acquisition.

Audiological Assessment

Audiological evaluation was performed to characterize the degree of hearing impairment and to exclude environmental causes of hearing loss. The assessment included auditory brainstem response (ABR) testing, distortion product otoacoustic emissions (DPOAE), tympanometry, and behavioral audiometry. ABR testing was conducted using Bio-logic auditory evoked potential equipment with ER-3A insert earphones. Behavioral and aided hearing assessments were performed using standard audiological protocols. Aided testing in the sound field was carried out using Phonak Naida S III UP or Phonak Supero 412 hearing aids depending on the patient.

### 2.3. Molecular Genetic Analysis

Peripheral blood samples (5–10 mL) were obtained from affected individuals and available family members. Genomic DNA was extracted from whole blood using the Gentra Puregene DNA extraction kit (Qiagen, Hilden, Germany) following the manufacturer’s protocol.

Whole-exome sequencing (WES) was performed to identify the underlying genetic cause of the disease as follows (1) The exome enrichment was performed using the SureSelect Human All Exon V6 capture kit (Agilent Technologies, Santa Clara, CA, USA). (2) The average sequencing depth achieved was ≥100× with greater than 95% of targeted bases covered at ≥20×. (3) Sanger sequencing was performed to confirm the identified variant (c.290T>G, p.Val97Gly) in all available family members, confirming co-segregation with the disease phenotype. Affected individuals were homozygous for the variant, while both parents were confirmed as heterozygous carriers in each family. Exonic regions and flanking splice junctions were enriched using a commercially available exome capture kit and sequenced via an Illumina high-throughput sequencing platform according to the manufacturer’s instructions. Sequence reads were aligned to the human reference genome (GRCh37/hg19) using the Burrows–Wheeler Aligner (BWA), and variant calling was performed using the Genome Analysis Toolkit (GATK) pipeline.

Detected variants were filtered and prioritized based on their location within coding regions or canonical splice sites, predicted functional impact on the protein sequence, and consistency with an autosomal recessive inheritance pattern. Variants with a minor allele frequency greater than 1% were excluded using population databases including dbSNP, the 1000 Genomes Project, the Exome Aggregation Consortium (ExAC), and the Genome Aggregation Database (gnomAD).

The potential pathogenicity of candidate variants was assessed using multiple in silico prediction tools including PolyPhen-2, SIFT, and MutationTaster. Variant interpretation and classification were performed according to the guidelines of the American College of Medical Genetics and Genomics (ACMG). Segregation analysis was performed in available family members to confirm the co-segregation of the identified variant with the disease phenotype.

## 3. Results

### 3.1. Clinical Findings

Both affected individuals demonstrated normal cognitive development and were alert and interactive during clinical evaluation. Patient 1 exhibited dysmorphic craniofacial features, including a long face, a high forehead, a short nose, low-set ears, and full lips. Patient 2 showed similar facial characteristics, with mild down-slanting palpebral fissures and thick lips.

Both patients presented with congenital sensorineural hearing loss and reported symptoms of nyctalopia (night blindness). Ophthalmic examination revealed pigmentary retinal changes consistent with retinal dystrophy. The detailed clinical characteristics of the affected individuals are summarized in Table 1.

**Figure 1:**
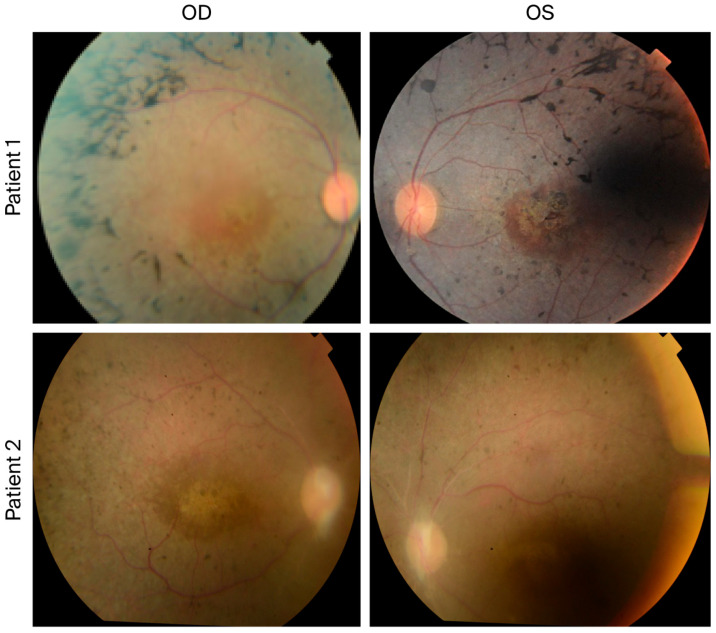
Fundus photographs of the two affected individuals carrying the PEX6 variant. Color fundus images demonstrate bilateral retinal pigmentary changes in both patients. The upper panels show the right eye (OD) and left eye (OS) of Patient 1, revealing scattered peripheral retinal pigment epithelium (RPE) pigmentary changes with areas of macular involvement. The lower panels show the OD and OS of Patient 2, demonstrating similar pigmentary alterations with attenuated retinal vessels and macular changes, consistent with retinal dystrophy in the spectrum of Heimler syndrome. Abbreviations: OD, right eye; OS, left eye; RPE, retinal pigment epithelium.

The retinal phenotype observed in our patients is consistent with previously reported ocular manifestations in HS, including pigmentary retinopathy and cone–rod dysfunction (Figure 1). Previous reports have demonstrated that *PEX6-*associated HS may present with macular dystrophy, highlighting retinal involvement as an important component of the disease spectrum.

### 3.2. Molecular Findings

Whole-exome sequencing (WES) was performed to identify the underlying genetic cause of the phenotype. Variant filtering prioritized rare homozygous variants consistent with an autosomal recessive mode of inheritance. This analysis identified a homozygous missense variant in the *PEX6* gene, c.290T>G (p.Val97Gly), in both affected individuals.

**Figure 2:**
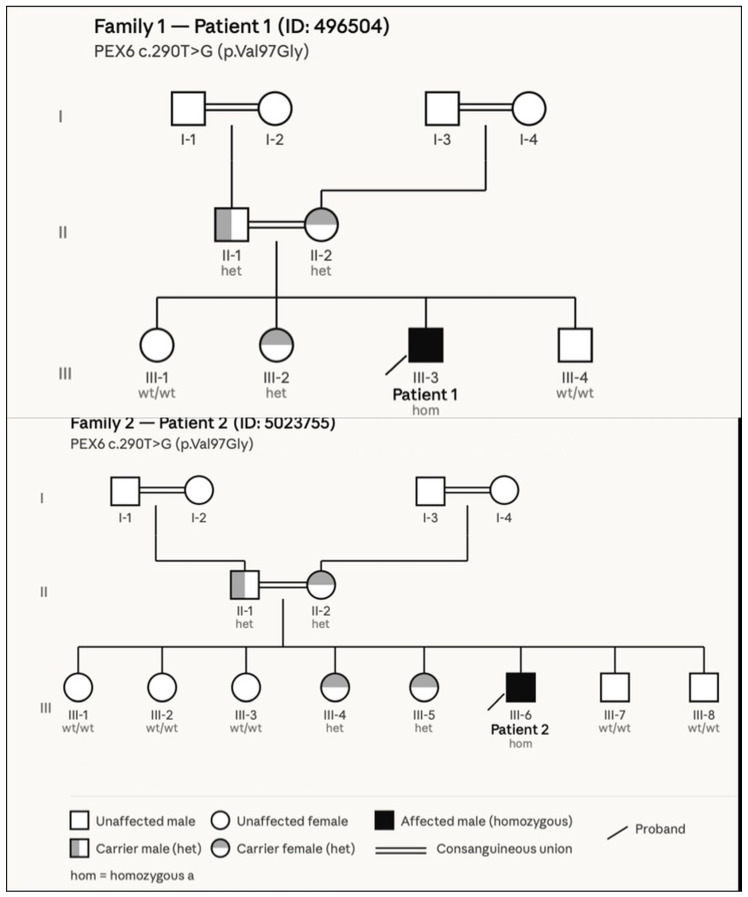
Pedigree analysis of two unrelated families with PEX6-associated Heimler syndrome. Pedigrees of Family 1 (top) and Family 2 (bottom) demonstrating segregation of the *PEX6* c.290T>G (p.Val97Gly) variant. In both families, the affected individuals (Patient 1: III-3; Patient 2: III-6) are homozygous for the variant (black symbols), while parents are heterozygous carriers (gray symbols), consistent with autosomal recessive inheritance. Unaffected are either heterozygous carriers or wild type (white symbols). Consanguinity is present in both families, as indicated by double lines. No affected individuals are reported in generations I or II. Squares represent males, circles represent females, and the diagonal arrow indicates the proband. wt/wt = wild type; het = heterozygous carrier; hom = homozygous.

Segregation analysis performed using Sanger sequencing confirmed that the variant co-segregated with the disease phenotype in both families, with the affected individuals being homozygous for the variant and unaffected parents being heterozygous carriers. The variant was absent from major population databases, including dbSNP, the 1000 Genomes Project, ExAC, and gnomAD, and it was also not detected in an internal Saudi exome database comprising approximately 3000 samples. In addition, multiple in silico prediction tools suggested that it had a deleterious effect on protein function. Taken together, these findings support the pathogenic role of the *PEX6* p.Val97Gly variant in the affected individuals. The variant has been submitted to ClinVar (Figure 2).

According to the American College of Medical Genetics and Genomics (ACMG) guidelines, the identified variant can be classified as likely pathogenic, based on its absence from population databases (PM2), its segregation with disease within the family (PP1), and computational evidence supporting a deleterious effect on protein function (PP3) [10]. Moreover, we have expanded the variant evidence as follows: (1) CADD score: the CADD phred score for p. Val97Gly is 28.4, exceeding the commonly used threshold of 20, consistent with a deleterious variant (PP3 further supported). (2) Conservation: as noted above, Val97 is conserved across vertebrate species (PM1 criterion located in a mutational hot spot or well-established functional domain). (3) Protein modeling: in silico structural modeling using AlphaFold2 predicts that the p. Val97Gly substitution disrupts local alpha-helical secondary structure in the N-terminal region of PEX6, potentially affecting protein folding and PEX1 interaction. (4) Updated ACMG criteria: based on the above, we now apply: PM1 (critical functional domain), PM2 (absent from population databases), PM3 (detected in trans with a pathogenic variant, if applicable), PP1 (co-segregation), PP3 (multiple computational tools predict deleteriousness, CADD > 20). This supports the likely pathogenic classification.

The identification of the same homozygous variant in two unrelated families may suggest a possible founder effect within the population, although further population-based studies are required to confirm this hypothesis.

Importantly, both patients were initially diagnosed with Usher syndrome, illustrating the diagnostic challenge posed by overlapping clinical features. Recognizing dental abnormalities such as amelogenesis imperfecta, along with molecular testing, is essential to distinguish HS from other inherited sensory disorders.

## 4. Discussion

Heimler syndrome (HS) is increasingly recognized as a distinct but mild phenotype within the spectrum of peroxisome biogenesis disorders. Since its initial clinical description, advances in molecular genetics have clarified that HS is primarily associated with hypomorphic variants in the peroxisome biogenesis genes *PEX1* and *PEX6*, which allow for the partial preservation of peroxisomal function and lead to a milder phenotype compared with classical Zellweger spectrum disorders [11]. In this study, we describe two unrelated Saudi patients with congenital sensorineural hearing loss and retinal dystrophy in whom whole-exome sequencing identified a homozygous missense variant in *PEX6*. Our findings further expand the mutational spectrum associated with *PEX6*-related Heimler syndrome and emphasize the importance of performing genomic testing in patients presenting with overlapping sensory phenotypes.

The clinical manifestations observed in our patients are consistent with previously reported cases of Heimler syndrome. Both individuals presented with congenital sensorineural hearing loss and retinal dystrophy manifested by nyctalopia and pigmentary retinal changes. Retinal involvement has been increasingly recognized as a major feature of HS, with many affected individuals demonstrating retinal degeneration resembling retinitis pigmentosa or cone–rod dystrophy [12]. Previous studies using multimodal retinal imaging have described characteristic findings including macular abnormalities, hyperautofluorescent deposits on fundus autofluorescence imaging, and the disruption of the photoreceptor ellipsoid zone identified via optical coherence tomography, reflecting progressive photoreceptor degeneration [12].

The molecular basis of Heimler syndrome is closely linked to the dysfunction of the peroxisomal protein import machinery. The PEX6 protein, a member of the AAA-ATPase family, forms a complex with PEX1 that is essential for recycling the PEX5 receptor during peroxisomal matrix protein import [13]. Disruption of this pathway impairs peroxisome biogenesis and alters several metabolic processes, including lipid metabolism and reactive oxygen species regulation. While severe loss-of-function variants in PEX6 are typically associated with classical Zellweger spectrum disorders, milder missense variants have been reported in individuals with Heimler syndrome, supporting the notion that residual protein activity determines disease severity [11,13].

The phenotypic overlap between Heimler syndrome and other inherited sensory disorders presents an important diagnostic challenge. In particular, the combination of congenital hearing loss and retinal dystrophy frequently raises suspicion for Usher syndrome, the most common cause of inherited deaf-blindness. However, several reports have demonstrated that patients initially suspected to have Usher syndrome were subsequently diagnosed with Heimler syndrome following next-generation sequencing analysis [14]. This highlights the importance of performing comprehensive genomic testing in patients with syndromic sensory impairment, particularly when clinical features are incomplete or atypical.

In recent studies, authors have also demonstrated that retinal involvement in Heimler syndrome may be more common than initially recognized. In several case series of molecularly confirmed HS, retinal dystrophy was present in the majority of affected individuals, although the age of onset and severity of visual impairment varied considerably [12,15]. This variability likely reflects differences in the functional consequences of specific *PEX* variants and the degree of residual peroxisomal activity. Long-term ophthalmologic monitoring is, therefore, recommended for affected individuals to detect progressive retinal degeneration and guide appropriate visual rehabilitation strategies.

An important clinical distinction between Heimler syndrome and Usher syndrome lies in the presence of dental abnormalities, particularly amelogenesis imperfecta, which is a hallmark feature of Heimler syndrome but is absent in Usher syndrome. In both patients described in this report, amelogenesis imperfecta was identified during clinical evaluation and served as a critical clue prompting molecular genetic testing beyond the Usher syndrome gene panel. Clinicians evaluating patients with congenital hearing loss and retinal dystrophy should therefore routinely include a dental examination as part of the diagnostic workup, as the presence of enamel hypoplasia or amelogenesis imperfecta may significantly narrow the differential diagnosis and guide appropriate genetic testing toward peroxisomal biogenesis disorders. This distinction has been well documented in the literature, where Heimler syndrome—caused by mutations in PEX1 or PEX6—is consistently associated with enamel defects, in contrast to Usher syndrome, which lacks systemic features outside the auditory and visual systems [3].

A broad differential diagnosis should be considered in patients presenting with combined sensorineural hearing loss and retinal dystrophy. Usher syndrome is the most common inherited cause of deaf-blindness and is caused by variants in genes such as *MYO7A* and *USH2A*; however, it is not associated with dental abnormalities. Alström syndrome, caused by variants in *ALMS1*, is characterized by cone–rod dystrophy, sensorineural hearing loss, obesity, and cardiomyopathy. Refsum disease, resulting from variants in *PHYH* or *PEX7*, presents with retinitis pigmentosa, hearing loss, cerebellar ataxia, and elevated plasma phytanic acid levels. In addition, *PEX1*-related peroxisomal disorders may clinically overlap with *PEX6-*related Heimler syndrome but are caused by variants in *PEX1* and may exhibit more severe peroxisomal dysfunction. Recognition of distinguishing features particularly the presence or absence of systemic findings such as amelogenesis imperfecta, metabolic abnormalities, or multisystem involvement is critical to guide targeted genetic testing and establish an accurate diagnosis [3,16,17,18].

The authors of several studies have described retinal degeneration and hearing loss in patients with Heimler syndrome caused by variants in *PEX1* or *PEX6* [11,12]. The clinical manifestations reported in these studies are largely consistent with the findings observed in our patients. A comparison of previously reported cases with those in this study is summarized in Table 2.

From a population genetics perspective, studies from regions with higher rates of consanguinity have played an important role in identifying novel pathogenic variants associated with rare autosomal recessive disorders. In Middle Eastern populations, large-scale genomic studies have demonstrated the effectiveness of exome sequencing in uncovering the genetic basis of previously undiagnosed inherited conditions [10]. The identification of additional *PEX6* variants in such populations contributes to growing understanding of the genetic diversity and clinical variability associated with Heimler syndrome.

## 5. Conclusions

In conclusion, in our study, we expand the clinical and molecular spectrum of *PEX6-*associated Heimler syndrome and underscore the importance of considering this condition in patients presenting with both congenital sensorineural hearing loss and retinal dystrophy. Early molecular diagnosis using next-generation sequencing technologies, particularly whole-exome sequencing, enables accurate clinical classification, facilitates appropriate genetic counseling, and improves recognition of the phenotypic variability associated with peroxisome biogenesis disorders. To the best of our knowledge, this is the first report describing the *PEX6* p.Val97Gly variant associated with Heimler syndrome in a Saudi population. The identification of this variant further broadens the mutational landscape of *PEX6* and highlights the diagnostic value of performing genomic testing in individuals presenting with Usher-like phenotypes. Our findings also emphasize the importance of considering Heimler syndrome in the differential diagnosis of patients with combined hearing loss and retinal dystrophy, particularly in populations with high rates of consanguinity, where rare autosomal recessive disorders may be enriched.

## 6. Limitations

This study has a key limitations including (1) the absence of functional assays to directly evaluate the impact of the variant on peroxisomal activity; (2) the lack of quantitative audiometric data (pure-tone audiograms); (3) the inability to obtain reliable OCT scans due to nystagmus; and (4) the absence of haplotype analysis to formally confirm or refute a founder effect; (5) Plasma very-long-chain fatty acids and other peroxisomal biomarkers were not measured in the studied patients. While peroxisomal metabolites including VLCFAs are typically normal or only mildly abnormal in Heimler syndrome, their measurement would have provided supporting biochemical evidence. Future studies should incorporate peroxisomal metabolic profiling as part of the diagnostic workup.

## Figures and Tables

**Table 1:** Detailed clinical features of the two affected individuals carrying the PEX6 variant identified in this study.

Patient Number	Patient 1 (496504)	Patient 2 (5023755)
Age	27	20
Gender/ethnicity	Male/Saudi	Male/Saudi
Family history	Six affected cousins (3 males and 3 females)	Three affected female cousins
Consanguinity	Parents are first-degree cousins	Parents are first-degree cousins
Ophthalmic assessments		
BCVA	OD: 20/200; OS: 20/150	OD: 10/400; OS: 20/800
Cycloplegic refraction	OD: +2 −1.50 ×90; OS: −0.50 −3.00 ×90	Not available
Cornea	Keratoconus	Clear
Iris	Normal OU	Normal OU
Lens	Lenticonus OU	OD: Cataractous; OD, OS: Pseudophakia
Optic disc	Waxy pallor OU	Waxy pallor OU
Retinal vessels	Attenuated vessels OU	Attenuated vessels OU
Retina	Scattered peripheral RPE bone corpuscles in the posterior pole with prominent macular defect OU	Minimal peripheral RPE bone corpuscles in the posterior pole with prominent macular defect OU
OCT	Not performed due to poor fixation	Not performed due to poor fixation
ERG	Abnormal photopic and normal scotopic	Abnormal photopic and normal scotopic
Systemic features		
Height	NA	182 cm
Weight	NA	117 kg
Occipitofrontal circumference	58 cm (98th centile)	57 cm (75th centile)
Face	Dysmorphic with long facies, high forehead, short nose, small low-set ears, and full lips	Dysmorphic with long flat facies, high forehead, mild down-slanting palpebral fissures, low-set mildly protruding ears, thick lips, and large saggy lower lips
Hearing	Bilateral sensory neural hearing loss	Bilateral sensory neural hearing loss
Nail	No clear sign of Beau’s line	Beau’s line in the middle finger of the hands
Teeth	Amelogenesis imperfecta	Amelogenesis imperfecta
Chest, Cardiovascular, Abdominal, CNS	Unremarkable	Unremarkable

Abbreviations: BCVA, best-corrected visual acuity; OD, right eye; OS, left eye; OU, both eyes; RPE, retinal pigment epithelium; OCT, optical coherence tomography; ERG, electroretinography; NA, not available; CNS, central nervous system.

**Table 2:** Reported *PEX6* variants associated with Heimler syndrome and their clinical characteristics compared with the present study.

Study	Gene	Variant	Protein Domain	Ethnicity	Hearing Loss	Retinal Findings	Dental Findings	Other Features
Ratbi et al., 2015 [3]	*PEX6*	p.Arg860Trp, p.Arg729Trp	AAA-ATPase domain	Moroccan	Congenital sensorineural hearing loss	Retinal dystrophy	Enamel hypoplasia	Nail abnormalities
Ratbi et al., 2015 [3]	*PEX6*	p.Arg601Gln	AAA-ATPase domain	European	Early-onset sensorineural hearing loss	Cone–rod dystrophy	Amelogenesis imperfecta	Mild ectodermal findings
Wangtiraumnuay et al., 2018 [5]	*PEX6*	Missense variants	AAA-ATPase domain	Thai	Sensorineural hearing loss	Macular dystrophy	Enamel defects	Nail changes
Gao et al., 2019 [11]	*PEX6*	p.Arg112His	AAA-ATPase domain	Chinese	Sensorineural hearing loss	Pigmentary retinopathy	Dental abnormalities	Variable ectodermal signs
Present study	PEX6	p.Val97Gly	AAA-ATPase- related region	Saudi Arabian	Congenital sensorineural hearing loss	Pigmentary retinal dystrophy	Amelogenesis imperfecta	Beau’s line in the middle finger of the hands in one patient

## Data Availability

The original contributions presented in this study are included in the article. Further inquiries can be directed to the corresponding author.
